# Can trained lay providers perform HIV testing services? A review of national HIV testing policies

**DOI:** 10.1186/s13104-016-2339-1

**Published:** 2017-01-04

**Authors:** David E. Flynn, Cheryl Johnson, Anita Sands, Vincent Wong, Carmen Figueroa, Rachel Baggaley

**Affiliations:** 1 Griffith University School of Medicine, Griffith University, Gold Coast, QLD Australia; 2 HIV Department, World Health Organization (WHO), Geneva, Switzerland; 3 Essential Medicines and Health Products, World Health Organization (WHO), Geneva, Switzerland; 4 Global Health Bureau: Office of HIV/AIDS, United States Agency for International Development (USAID), Washington, DC USA; 5 8 Bellevue St, Chatswood West, NSW 2067 Australia

**Keywords:** HIV testing, Lay providers, Community health workers, HIV policy

## Abstract

**Background:**

Only an estimated 54% of people living with HIV are aware of their status. Despite progress scaling up HIV testing services (HTS), a testing gap remains. Delivery of HTS by lay providers may help close this testing gap, while also increasing uptake and acceptability of HIV testing among key populations and other priority groups.

**Methods:**

50 National HIV testing policies were collated from WHO country intelligence databases, contacts and testing program websites. Data regarding lay provider use for HTS was extracted and collated. Our search had no geographical or language restrictions. This data was then compared with reported data from the Global AIDS Response Progress Reporting (GARPR) from July 2015.

**Results:**

Forty-two percent of countries permit lay providers to perform HIV testing and 56% permit lay providers to administer pre-and post-test counseling. Comparative analysis with GARPR found that less than half (46%) of reported data from countries were consistent with their corresponding national HIV testing policy.

**Conclusions:**

Given the low uptake of lay provider use globally and their proven use in increasing HIV testing, countries should consider revising policies to support lay provider testing using rapid diagnostic tests.

**Electronic supplementary material:**

The online version of this article (doi:10.1186/s13104-016-2339-1) contains supplementary material, which is available to authorized users.

## Background

Despite achievements and expansion of HIV testing services (HTS) to date, presently only 54% of people living with HIV are aware of their serostatus [1]. It is estimated that there are approximately 2 million new infections annually, half of which are among key populations who often have poor access to HTS, and when they do test, they test late in their infection [2]. Additional and more focused HTS approaches are critical to reach the United Nations “90-90-90” global targets which aims to diagnose 90% of all people living with HIV by 2020, have 90% of those diagnosed receiving sustained antiretroviral therapy (ART) and aiming to have 90% of those on ART to be virally suppressed [3]. To reach this testing target of 90%, it is critical to address the existing gaps in service provision, including health worker shortages and the lack of community-based HTS, which limit the expansion and effectiveness of HTS in many resource-limited settings [4–6].

Testing for HIV infection is a crucial part of HTS. Historically, HIV testing involved lengthy laboratory testing of blood collected by venipuncture for HIV antibodies involving specialist equipment and specialist staff. Rapid diagnostic tests (RDTs) for HIV were introduced in the early 2000’s and allow for testing of fingerstick whole blood or oral fluid, with a result established in under an hour. Due to their low cost, reliability, ease of use and speed, they are now widely used as a diagnostic means (within a testing algorithm) by low resource countries. Testing with RDTs (both fingerstick blood and oral fluid rapid tests) is possible with adequately trained lay providers. Trained lay providers have been delivering HTS for decades and in many settings [7] across the Americas [8], Europe [9, 10], sub-Saharan Africa [11–17] and Asia [18]. Task-sharing—the rational redistribution of tasks from “higher-level” cadres of health professionals to trained lay provider cadres—can expand HTS more broadly, has been shown to increase uptake of HIV testing [8, 12, 19], and can facilitate testing in community based and outreach services, especially for key populations [20]. Lay providers have been shown to provide high quality services, provide accurate HIV test results [11, 18], and may also be lower in cost than services performed by other health providers.

Since 2008 the World Health Organization (WHO) has recommended task-sharing clinical services within the HIV continuum of diagnosis, prevention, care and treatment [21]. In particular, WHO recommends that programs increase the scope of work of trained lay providers and introduce task-sharing across professional cadres [22]. This task-sharing involves providing clinical services, counseling and referrals to HIV specialist services. However, in many settings lay provider HTS has not been utilized and suboptimal HTS coverage persists.

Global AIDS Response Progress Reporting (GARPR) (WHO, UNAIDS, UNICEF) is an annual initiative that allows representatives from government health departments to voluntarily report information such as epidemiological statistics as well as current clinical HIV testing practices within their country. This information is based upon the clinical practices within the country. According to data from the 2015 Global AIDS Response Progress Reporting (GARPR) (WHO, UNAIDS, UNICEF), only 52% (n = 65/124) of reporting countries said that lay providers were allowed to perform HIV rapid diagnostic tests (RDTs) [23]. With this low reported uptake of lay provider use, there is no data regarding the role of HIV national health policies in lay provider utilisation and as a result these policies could be a key barrier to lay provider delivered HTS.

Within this paper we analyzed national HIV testing policies from a range of countries to determine the role of lay providers in delivering HIV testing (through the use of RDTs) as well as pre and post-test counselling. In doing so, we sought to investigate whether national health policy permitted trained lay providers to perform HTS (specifically RDTs and counselling) and compare this to the GARPR reported data to determine whether the national HIV health policy reflects what happens in HIV healthcare systems.

## Methods

### Policy criteria

We collected national policy documents from WHO country intelligence databases, national HIV testing program websites and comprehensive web-based searching through databases and national health department websites. The search and analysis of national HIV policies took place from November 1, 2014 to December 21, 2014. Our search had no geographical or language restrictions. We selected the most recent HIV national testing policies and, when not available, national policy statements on who can perform HTS. We excluded HTS standards and protocols developed by nongovernmental organizations, donors or other technical agencies.

### Policy analysis

We reviewed the policies in full text, extracting three pieces of information: whether trained lay providers can perform HIV rapid tests using finger stick blood, whether trained lay providers can perform HIV rapid tests using oral fluid and whether trained lay providers can perform pre and post-test counselling (which includes the delivering of results). Six researchers (VW, CB, AS, RB, CJ and DF) developed the data extraction form. One reviewer (DF) extracted data, two other reviewers (RB and CJ) evaluated and approved the extracted data. Disagreements between reviewers (RB, CJ and DF) were resolved through discussion and consensus. Our results were then compared with data reported by countries to GARPR (WHO, UNAIDS, UNICEF) as of July 1, 2015, based on the policy and programmatic indicator that asks whether current HIV testing guidelines recommend that RDTs can be performed by lay providers [24].

### Global AIDS response progress reporting (GARPR) data

The GARPR online reporting tool is a method of collection of clinical data and epidemiological statics from a variety of countries on various indicators of global HIV response. Primarily low- and middle-income countries report this data to WHO, UNAIDS and UNICEF. These indicators include epidemiological HIV data as well as a variety of questions regarding HIV practices. Specifically, there are four primary questions about national HIV testing in the GARPR online reporting tool, of which lay provider testing with RDTs is a sub-question. The data is a reflection of the clinical activities that occur within that country (i.e. a reflection of what occurs ‘on the ground’), which should ideally be reflected in national HIV policies, however sometimes this is not the case.

National representatives from reporting countries are responsible for providing this information according to the guidance provided by WHO, UNAIDS and UNICEF [24]. These representatives are designated individuals from governmental health departments or government HIV-specific programs

Data regarding whether lay providers can administer HTS in each country was collected in July 2015 and compared to the same information extracted from their corresponding national HIV testing policies.

The type of HIV testing allowed (i.e. fingerstick or oral fluid based) to be performed by lay providers was not specified in the question the GARPR data collection tool.

## Results

We identified HIV policies from 61 countries and excluded policies from 11 countries because they did not include sufficient information, were not official documents or were not able to be translated. Thus, we included a total of 50 national testing polices in this review: 25 countries were from the WHO African region, four countries from the WHO American region, nine from the WHO Eastern-Mediterranean region, three from the WHO European region, three from the WHO Southeast Asia region, and six from the WHO Western Pacific region.

### Analysis of RDTs (using fingerstick blood) use by lay providers

**Fig. 1:**
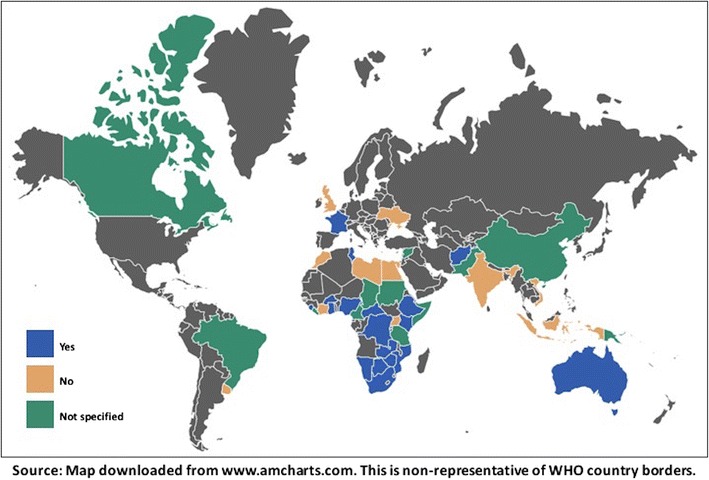
Distributional map showing whether national HIV testing policies permit lay providers to perform RDTs using fingerstick/whole blood (n = 50)

Twenty-one of 50 national policies allow lay providers to use RDTs using fingerstick blood and 15 of 50 country policies explicitly prohibit lay providers from performing them. Fourteen of 50 countries do not specify a role for lay providers in HIV testing with fingerstick RDTs (Fig. 1).

### Analysis of RDTs (using oral fluid) use by lay providers

Nine out of the 50 countries had policies allowing lay providers to use RDTs using oral fluid while ten countries specifically did not permit lay providers to use oral fluid RDTs. The majority of countries (n = 31/50) did not specify if lay providers could use RDTs using oral fluid or not.

### Analysis of fingerstick blood vs oral fluid RDT use by lay providers

Countries that do not permit lay provider RDT testing (with finger stick blood) either do not allow oral fluid testing as well or do not specify if they can perform oral fluid based RDTs. The number of countries that did not allow lay providers to perform either type of RDT was evenly spread across all geographical areas, with no obvious predisposing factor as to why those countries do not permit lay provider testing. Reasons behind the non-use of lay providers were not addressed within the national policies.

### Analysis of pre and post-test counselling by lay providers

**Fig. 2:**
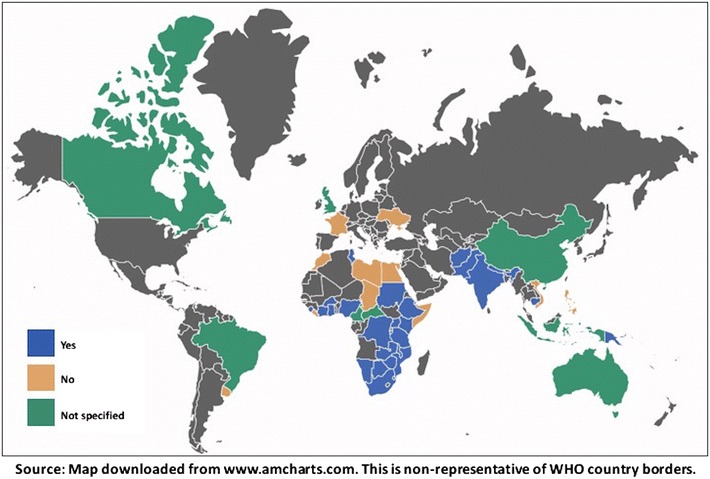
Distributional map showing whether national HIV testing policies permit lay providers to perform pre and post-test counseling (n = 50)

Twenty-eight of 50 countries permit lay providers to provide pre- and post-test counseling, 12 of 50 countries prohibit lay providers from performing counseling and 10 of 50 countries did not specify in their policies the role of lay providers in administering counseling (Fig. 2).

### Sub-analysis of WHO African region HIV testing policies

The majority of countries from the WHO African region allow lay providers to perform HIV RDTs using fingerstick/whole blood (n = 16/25) with four countries not allowing lay providers to perform RDTs with fingerstick blood and five countries not specifying the information. Further analysis on the utilization of RDTs using oral fluid shows that only one-fifth (n = 5/25) of countries in the WHO African region permit lay providers to perform RDTs using oral fluid with three countries not permitting it and 17 of 25 countries not specifying if lay providers can perform RDTs using oral fluid. The WHO African region has the largest percentage of countries per region (80%) that allow lay providers to perform HIV pre- and post-test counseling (n = 20/25).

### GARPR 2014–2015 data

A comparison between reviewed national policies and GARPR reporting showed that in 12 of the 50 countries analyzed the national HIV policy and the GARPR data disagreed regarding whether lay providers could perform HIV testing. Of the 12 policies, five countries reported through GARPR that lay providers cannot perform HTS using RDTs, but their national policy stated that they could. Additionally, seven reviewed policies stated that lay providers were not allowed to provide HTS using RDTs, but according to GARPR reporting they could perform them.

In 23 of 50 countries the HIV testing policies had information that matched the reported GARPR data and 15 of 50 either had HIV testing policies or GARPR data that did not specify the role of lay providers.

GARPR data is not collected on whether lay providers can perform pre and post-test counselling. We were therefore unable to compare our collected data to any reported data.

### Raw data

For further analysis, a table with the raw data of each country is provided as an additional file to this article. It can also be found at: https://figshare.com/s/a89c08bc4819d5e47db6

The table outlines each country and whether their policy provides lay providers to perform each of the HTS (finger stick blood RDT, oral fluid RDT and pre and post test counselling).

## Discussion

This is the first review of national policies on HTS for inclusion of lay providers. Similar reviews have been undertaken on HIV policy regarding self-testing [25], but to date there is no information regarding the role of lay providers in national HIV policies.

This review revealed that of the 50 countries analyzed, 58% either do not permit lay providers to perform HIV RDTs using fingerstick blood (the most common type of HIV RDT) or do not specify if they can, while 44% do not permit or do not specify whether lay providers can perform HIV pre- and post-test counseling. The regional comparison analysis showed that the WHO African region had the highest proportion of countries in which lay testing is permitted.

Thirteen of the 50 reviewed policies did not specify or explicitly outline the role of lay providers in providing HTS. While some of these countries may have provisions that allow lay providers to perform HIV testing and pre- and/or post-test counseling, the lack of explicit reference to the role of lay providers may introduce misunderstandings or misinterpretations by providers and national program managers. Furthermore, if lay testing is not explicitly sanctioned, systems to support training, supervision and provide quality assurance of lay testing may not be routinely and consistently applied. Countries should regularly review their national HIV testing policy to make sure it contains correct information, reflects national practice as well as lay provider training, testing and quality assurance.

The difference between reported GARPR data and that extracted from official HIV testing policies may be due to several reasons such as policies that had not been updated to account for lay provider use, incorrect information from the national representative regarding lay provider use in their country, or the policy may not have been clear regarding the role of lay providers in HIV testing.

GARPR data only asks if lay providers are permitted to perform RDTs in general and does not specify whether lay providers are permitted to perform the different types of RDTs (i.e. fingerstick whole blood or oral fluid). This specific information would have helped to give greater clarity on the role of lay providers and aided in our comparison of GARPR data to national HIV policy.

Of the 12 countries with differing information, half (n = 6/12) of them were from African nations. This difference demonstrates a disparity between reported data and national policy information. Greater care must be taken when reporting GARPR data to make sure it correlates with approved practice. It is suggested that in a bid to increase accuracy, the reporting of GARPR data could be accompanied by policy documents to support their reported data and help catalyze regular reviewing of national policy documents.

Through task-sharing, lay provider HTS have been widely implemented throughout Africa. Sub-analysis of data from the WHO African region suggests that there is a more supportive policy environment for lay provider HTS compared to other global regions. Task sharing to increase the scope of work for lay providers is important to scaling up HTS, as recent reviews have shown that it is acceptable, can be low cost and can increase uptake of HTS, particularly among key populations who are generally underserved [26]. Thus, based on the review of existing evidence, WHO recommends that trained lay providers can perform RDTs safely and effectively [26].

### Limitations

Although we reviewed the most currently available HTS policies, some may be out of date or in the process of being updated. As we collected and analyzed national testing policies, it is possible that some information on lay provider testing was not specified in these, but instead included in national PMTCT guidelines, treatment guidelines or other national guidelines.

Although 50 national policies were collected, this did not match the number of countries reported in GARPR data (n = 124). A greater number of HIV testing policies would have made for a greater comparison and stronger analysis however this difference was explained by the difficulty in obtaining formal HIV policies, the lack of formal HIV policies from a number of the reporting countries and the time limitations that were placed on the analysis.

Data on whether lay providers are permitted to perform pre and post-test counseling was not collected as a part of the 2014–2015 GARPR reporting cycle. Therefore, we could not compare information from reviewed national policies with GARPR reports. Inconsistencies identified between national policies and GARPR reports highlight that the term lay provider may need further definition. It is possible that errors in reporting or missing information may have occurred because of differences in terminology.

## Conclusion

To increase access to and effectiveness of HTS, WHO supports the use of trained lay providers for HIV testing. Trained lay testers can have an important role in providing services in communities to people who do not attend health facilities. Many countries continue to prohibit testing by trained lay testers, and this may be a barrier to reaching those most vulnerable to HIV who do not access existing services.

For countries to increase reach of their HTS, task sharing to lay providers must be a part of a country’s testing protocol and should be included in national HIV testing guidelines. Lay provider use is an important way to provide acceptable services to key populations, and those other populations who might not present for testing using traditional laboratory-based HTS. Ensuring that RDTs using oral fluid and fingerstick whole blood can be conducted by lay providers will remain the cornerstone of HTS and will be necessary to promote the expansion and uptake of HIV testing.

National representatives must also take greater care when reporting on national HIV testing practices, ensuring that the data reported is up-to-date and reflected in national policies. Regular updating of policies with regards to all aspects of HTC is encouraged.

Increased efforts are needed to accelerate policy adaptation and implementation of the new 2015 WHO *Consolidated guidelines on* HTS, to help expand HIV testing coverage in areas and among populations with the greatest need.

## Additional file

**Additional file 1.** Annex 1: Overview of the roles of lay providers according to national HIV testing. Description of Data: A table demonstrating each individual countries use of lay providers in HIV testing for different modalities and HIV pre and post test counselling.

## Abbreviations

HTS: HIV testing services
GARPR: global AIDS response progress reporting
WHO: World Health Organisation
UNAIDS: The Joint United Nations Programme on HIV and AIDS
UNICEF: The United Nations Children’s Fund
RDT: rapid diagnostic test
