# Supplementary material for: Can trained lay providers perform HIV testing services? A review of national HIV testing policies
Source: BMC Res Notes. 2017 Jan 4;10:20. doi: 10.1186/s13104-016-2339-1 (PMC5216526; doi:10.1186/s13104-016-2339-1)
Supplement: Supplementary file 1 — Additional file 1. Annex 1: Overview of the roles of lay providers according to national HIV testing. Description of Data: A table demonstrating each individual countries use of lay providers in HIV testing for different modalities and HIV pre and post test counselling. [file 13104_2016_2339_MOESM1_ESM.pdf]

Annex I: Overview of the roles of lay providers according to national HIV testing policies (n=50)

General			Can lay providers perform:		
WHO region	Country	Document reference	Fingerstick whole blood-based RDTs	Oral fluid-based RDTs	Pre- and post-test counseling
African region	Burundi	(1)	NS	NS	Y
	Botswana	(2)	Y	NS	Y
	Burkina Faso	(3)	Y	NS	Y
	Cameroon	(4)	NS	NS	NS
	Central African Republic	(5)	Y	NS	NS
	Chad	(6)	NS	NS	N
	Comoros	(7)	N	NS	Y
	Cote d'Ivoire	(8)	N	N	Y
	Democratic Republic of the Congo	(9)	Y	NS	Y
	Ethiopia	(10)	Y	Y	Y
	Ghana	(11)	Y	Y	Y
	Kenya	(12)	Y	Y	Y
	Liberia	(13)	NS	NS	N
	Malawi	(14)	Y	NS	Y
	Mozambique	(15)	Y	NS	Y
	Namibia	(16)	Y	Y	Y
	Nigeria	(17)	Y	NS	Y
	Rwanda	(18)	N	N	N
	Sierra Leone	(19)	Y	NS	Y
	South Africa	(20)	Y	NS	Y
	Swaziland	(21)	Y	NS	Y
	United Republic of Tanzania	(22)	NS	NS	Y
	Uganda	(23)	N	N	Y
	Zambia	(24)	Y	NS	Y
	Zimbabwe	(25)	Y	Y	Y
Americas region	Brazil	(26)	NS	Y	NS
	Canada	(27)	NS	NS	NS
	Trinidad and Tobago	(28)	NS	NS	NS
	Uruguay	(29)	N	N	N
Eastern Mediter-ranean region	Afghanistan	(30)	Y	Y	Y
	Egypt	(31)	N	N	N
	Libya*	(32)	N	NS	N
	Morocco*	(33)	N	N	N
	Pakistan	(34)	NS	NS	Y
	Somalia	(35)	NS	NS	N
	Sudan	(36)	NS	NS	Y
	Syria*	(37)	NS	NS	NS
	Tunisia	(38)	Y	NS	Y
European region	France	(39)	Y	Y	N
	Ukraine	(40)	N	NS	N
	United Kingdom	(41)	N	N	NS
South-East Asia region	India	(42)	N	N	Y
	Indonesia	(43)	N	NS	NS
	Nepal	(44)	N	N	Y
Western Pacific region	Australia	(45)	Y	Y	NS
	Cambodia	(46)	Y	NS	Y
	China	(47)	NS	NS	NS
	Papua New Guinea	(48)	NS	NS	Y
	Philippines	(49)	N	N	N
	Viet Nam	(50)	N	NS	N

Key	
Y	Yes
NS	Not specified in policy
N	No
*	Denotes the use of consultation with a key expert

References:

[1] Guide de Conseil en Matiere de VIH/SIDA/IST. Gouvernement de la Republique du Burundi: Ministère de la Santé Publique du Burundi; 2004.

[2] National Guidelines: HIV Testing and Counselling. Republic of Botswana: Ministry of Health; 2009.

[3] Normes et Directives Nationales de Conseil Depistage de L'infection a VIH au Burkina Faso. Gouvernement de Burkina Faso Government: Ministere de la Sante; 2008.

[4] Directives Nationales de prevention et de prise en charge du VIH au Cameroun. Gouvernement de la République du Cameroun: Ministere de Sante Publique; 2014.

[5] Guide du depistage et conseil du VIH a l'initiative du prestataire. Gouvernement de la Republic Centrafricaine: Ministere de la Sante Publique; 2010.

[6] Manuel de Formation en Counseling VIH/SIDA/IST. Republic du Tchad: Ministere de la Sante Publique; 2011.

[7] Guide de prise en charge de l'infection à VIH Aux Comores. Union des Comores: Ministère de la Santé, Programme National de lutte contre le SIDA; 2007.

[8] Document de Normes et Directives Nationales du Conseil et Depistage Volontaire du VIH en Cote D'Ivoire. Abidjan, Republic de Cote D'Ivoire: Le gouvernement de Cote D'Ivoire: Ministere de la Solidarite, de la Sante et de la Securite Sociale; 2002.

[9] Normes et directives en conseil et depistage volontaire du VIH/SIDA. Gouvernement de la République démocratique du Congo: Ministere de la Santé/ Programme national de lutte contre le SIDA et les IST; 2004.

[10] Guidelines for HIV Counselling and Testing in Ethiopia. Government of the Federal Democratic Republic of Ethiopia: Federal Ministry of Health, Federal HIV/AIDS Prevention and Control Office; 2007.

[11] National guidelines for the implementation of HIV counseling and testing in Ghana. Government of the Republic of Ghana; 2008.

[12] National Guidelines for HIV Testing and Counselling in Kenya. Government of Kenya: Ministry of Public Health and Sanitation; 2010.

[13] Integrated Guidelines for Prevention, Testing, Care and Treatment of HIV/AIDS in Liberia. Government of the Republic of Liberia: Ministry of Health and Social Welfare & National AIDS and STI Control Program; 2007.

[14] Malawi Comprehensive HIV Testing and Counselling training. Government of Malawi: Ministry of Health; 2013.

[15] Guião Estratégico-Operacional para Implementação das Unidades de Aconselhamento e Testagem em Saúde (UATS). República de Moçambique: Ministério da Saúde; 2008.

[16] National Guidelines for HIV Counselling and Testing in Namibia. Government of the Republic of Namibia: Ministry of Health and Social Services; 2011.

[17] National Guidelines for HIV Counselling and Testing. Government of Nigeria: Federal Ministry of Health; 2011.

[18] National Guidelines for Prevention and Management of HIV, STIs & Other Blood Borne Infections. Government of the Republic of Rwanda: Ministry of Health; 2013.

[19] Guidelines For Voluntary Confidential Counselling and Testing In Sierra Leone. Government of the Republic of Sierra Leone: Ministry of Health and Sanitation; 2003.

[20] HIV Counselling and Testing (HCT) Policy Guidelines. Government of the Republic of South Africa: Department of Health; 2011.

[21] HTC Program Annual Report 2012. Kingdom of Swaziland: Ministry of Health; 2012.

[22] National guidelines for the management of HIV and AIDS. The government of the United Republic of Tanzania: Ministry of Health and Social Welfare; 2012.

[23] National Implementation Guidelines for HIV Counselling and Testing in Uganda. Government of the Republic of Uganda: Ministry of Health; 2010.

[24] National Guidelines for HIV Counselling & Testing. Government of the Republic of Zambia: Ministry of Health 2006.

[25] National Guidelines on HIV Testing and Counselling. Government of the Republic of Zimbabwe: Ministry for Health and Child Care; 2014.

[26] Manual Técnico Para O Diagnóstico Da Infecção Pelo HIV. Government of Brazil: Department of Health; 2014.

[27] Human Immunodeficiency Virus: HIV Screening and Testing Guide. Ottowa, Canada: Centre for Communicable Diseases and Infection Control, Public Health Agency of Canada; 2012.

[28] National HIV testing and Counselling Policy. Government of the Republic of Trinidad and Tobago: Ministry of Health; 2012.

[29] Infección por virus de la inmundodeficiencia humana (VIH-SIDA)- Guías para diagnóstico, tratamiento antiretroviral y monitorización adultos y embarazadas. Ministerio de Salud Pública, La Organización Panamericana de la Salud; 2006.

[30] National Guidelines of HIV Testing and Counseling Services. Islamic Republic of Afghanistan: Depatment of Public Health; 2013.

[31] National Guidelines for Voluntary HIV Counseling and Testing. Arab Republic of Egypt: Ministry of Health and Population/ National AIDS Program; 2004.

[32] El-Gueneidy M, Bahaa T. HIV Voluntary Counseling and Testing Services: A Guidelines for Service Providers (Libya). 2010.

[33] Morocco HIV Testing Information provided by Dr Mehdi Karkouri 2014.

[34] HIV Voluntary Counselling and testing (VCT) Guidelines for Pakistan. Government of Pakistan; 2010.

[35] HIV Testing and Counseling in Health Care Settings. Government of the Federal Republic of Somalia; 2011.

[36] Guidelines for Voluntary Counselling and Testing (VCT). Government of the Republic of Sudan: Ministry of Health; 2008.

[37] Syria HIV testing information provided by WHO EMRO contact Joumanna Hermez. 2014.

[38] Stratégie nationale de dépistage de l'infection à VIH. Government of the Tunisian Republic: Ministry of Health; 2014.

[39] Dépistage de l'infection par le VIH en France: Stratégies et dispositif de dépistage. Collège de la Haute Autorité de Santé; 2009.

[40] Protocol of Voluntary HIV Counseling and Testing. Government of Ukraine: Ministry of Health (Decree #415); 2005.

[41] UK Standards for Microbiology Investigations: Anti-HIV Screening. Standards Unit, Microbiology Services, Public Health England; 2014.

[42] Guidelines on HIV Testing. Republic of India: Ministry of Health and Family Welfare/ National AIDS Control Organisation 2007.

[43] Pedoman Pelaksanaan Konseling Dan Tes HIV. Peraturan Menteri Kesehatan Republik Indonesia; 2014.

[44] National Guidelines for Voluntary HIV/AIDS Counseling and Testing. Government of Nepal: Ministry of Health and Population; 2007.

[45] 2014 National HIV Testing Policy. Australian Government: Department of Health and Ageing; 2014.

[46] Standard Operating Procedures for HIV Testing and Counseling (HTC). Kingdom of Cambodia: Ministry of Health/ National Center for HIV/AIDS, Dermatology and STDs; 2012.

[47] National Guidelines for Detection of HIV/AIDS. People's Republic of China; Chinese Center for Disease Control and Prevention; 2009.

[48] National Guidelines for HIV Counselling and Testing in Papua New Guinea. Government of Papua New Guinea: Department of Health; 2010.

[49] Administrative Order: 2010- 0028. Policies and Guidelines in the conduct of Human Immunodeficiency Virus (HIV) Counseling and Testing in Community and Health Facility Settings. Government of the Republic of the Philippines: Department of Health; 2010.

[50] Decision on promulgation of voluntary HIV counseling and testing (VCT) guidelines. Government of the Socialist Republic of Vietnam: Ministry of Health; 2007
